# Feasibility of Total Neoadjuvant Treatment Using Short-Course MRI-Guided Radiotherapy with an Integrated Boost in Locally Advanced Rectal Cancer: A Pilot Study †

**DOI:** 10.3390/cancers18132024

**Published:** 2026-06-23

**Authors:** Koen Kortbeek, Amy De Haar-Holleman, Jacques Bodenstein Bezuidenhout, Ellen Van Eetvelde, Sven Van Laere, Thierry Gevaert, Alexandra Sermeus, Benjamin Vanspeybroeck, Guy Soete, Mark De Ridder

**Affiliations:** 1 Department of Medical Oncology, Universitair Ziekenhuis Brussel, Laarbeeklaan 101, 1090 Brussels, Belgium; amy.dehaarholleman@uzbrussel.be; 2 Faculty of Medicine and Pharmacy, Vrije Universiteit Brussel (VUB), Universitair Ziekenhuis Brussel (UZ Brussel), Translational Oncology Research Center (TORC), Team Laboratory for Medical and Molecular Oncology (LMMO), Laarbeeklaan 103, 1090 Brussels, Belgium; 3 Department of Radiotherapy, Universitair Ziekenhuis Brussel, Laarbeeklaan 101, 1090 Brussels, Belgium; jacquesbodenst.bezuidenhout@uzbrussel.be (J.B.B.); sven.vanlaere@uzbrussel.be (S.V.L.); thierry.gevaert@uzbrussel.be (T.G.); benjamin.vanspeybroeck@uzbrussel.be (B.V.); guy.soete@uzbrussel.be (G.S.);; 4 Department of Abdominal Surgery, Universitair Ziekenhuis Brussel, Laarbeeklaan 101, 1090 Brussels, Belgium; ellen.vaneetvelde@uzbrussel.be; 5 Department of Gastroenterology, Universitair Ziekenhuis Brussel, Laarbeeklaan 101, 1090 Brussels, Belgium; alexandra.sermeus@uzbrussel.be

**Keywords:** locally advanced rectal cancer, MRI-guided radiotherapy, short-course radiotherapy, total neoadjuvant therapy

## Abstract

**Background**: The RAPIDO trial demonstrated that short-course radiotherapy (5 × 5 Gy) followed by chemotherapy and surgery decreased disease-related treatment failure compared to long-course chemoradiotherapy. However, at 5-year follow-up, an increase in locoregional recurrence was observed. A strategy to improve local control is to increase radiotherapy dose. We present a retrospective analysis of a patient cohort treated with intensified MRI-guided radiotherapy. **Methods**: Between November 2021 and January 2024, 28 patients with cT3-4 N0-2 rectal cancer were included. Treatment consisted of adaptive MRI-guided external beam radiotherapy 5 × 5 Gy on the mesorectum with a simultaneous integrated boost (SIB) of 5 × 6 Gy on the gross tumor volume (GTV) followed by chemotherapy consisting of 18 weeks of CAPOX or FOLFOX. In case of a complete clinical response (cCR) following neoadjuvant treatment, a watchful-waiting approach was considered. The primary outcome was local and distant recurrence. **Results**: 27 patients were included in the analysis, with a median follow-up of 39 months. One patient was excluded due to loss to follow-up. Six patients had a pathological complete response (pCR) on surgery, and seven in the watch-and-wait protocol had persistent cCR. Locoregional failure was observed in one patient and four patients developed distant metastasis, leading to death in one patient. **Conclusions**: Total neoadjuvant treatment (TNT) in locally advanced rectal cancer using an MRI-guided integrated boost on the GTV is tolerable and resulted in a high rate of pCR (22%) and persistent cCR (26%), with limited locoregional failure (4%) and distant relapse (15%).

## Simple Summary

This study explores an MRI-guided total neoadjuvant treatment (TNT) approach for locally advanced rectal cancer that aims to increase tumor response while preserving organ function. In a small, single-center cohort, nearly half of the patients achieved a complete response, with low rates of local and distant recurrence over more than three years of follow-up. These findings suggest that intensifying radiotherapy with an MRI-guided boost, followed by systemic chemotherapy, may offer higher chances of tumor eradication and organ preservation than standard TNT regimens while maintaining acceptable toxicity. Although the retrospective design and limited sample size mean that the results should be interpreted with caution, they highlight a potentially promising strategy that merits confirmation in larger, prospective studies.

## 1. Introduction

When first reported by Bahadoer et al., the RAPIDO trial demonstrated that short-course radiotherapy (SC-RT) followed by chemotherapy and surgery decreased disease-related treatment failure at three years compared to long-course radiotherapy (LC-CRT) in patients with locally advanced rectal cancer [hazard ratio (HR) 0.75, *p* = 0.019] [1].

The initial results of RAPIDO led to widespread adoption of this treatment protocol. However, an increased rate of local recurrence was later reported in the intervention arm, leading to a decreased implementation of this regimen [2]. Analysis of the RAPIDO data has shown that the pathological complete response (pCR) predicts the local control rate in the total neoadjuvant treatment of locally advanced rectal cancer [3]. This leads to the suggestion that the higher rate of locoregional failure in RAPIDO is driven by those patients who respond insufficiently to radiotherapy.

In our hospital, we had some reservations concerning the biological effective dose (BED) of SC-RT compared to LC-CRT. Considering an *α*/*β* ratio of 10 Gy for rectal cancer, the BED_10_ of 5 × 5 Gy is 37.5 Gy (EQD_2_ 31.3 Gy). Using the LQ model on commonly used long-course RT schemes—like 25 × 1.8 Gy, 25 × 2 Gy, or 28 × 1.8 Gy—the tumor BED_10_ ranges from 53.1 to 60 Gy (EQD2 44.2–50 Gy) [4]. Although the accelerated nature of SC-RT allows for a lower BED due to the reduced time for repopulation compared to LC-CRT, we opted for a slightly adapted treatment regimen that includes a simultaneous integrated boost to the gross tumor volume (GTV). The simultaneous integrated boost (5 × 6 Gy to the GTV) yields a BED_10_ of 48.0 Gy (EQD_2_ 40.0 Gy), which remains lower than a typical LC-CRT BED but higher compared to SC-RT schedules such as the one used in the RAPIDO trial.

The introduction of this treatment protocol coincided with the adoption of MRI-guided radiotherapy (MRgRT) at our institution. MRgRT has several advantages over standard CT-guided radiotherapy in rectal cancer. MRI offers superior soft-tissue contrast, real-time imaging and gating, which allow for smaller margins and dose escalation without increasing the normal tissue complication probability [5,6]. In this paper, we report the observations of combining short-course MRgRT and a simultaneous integrated boost. Preliminary results from this dataset were presented as a poster at the ESTRO 2025 Annual Meeting [7].

## 2. Materials and Methods

### 2.1. Patient Selection

For this retrospective analysis, we included patients with locally advanced rectal cancer treated between July 2021 and January 2024. Inclusion criteria were age > 18 years, ECOG performance status 0–1, adenocarcinoma within 15 cm of anal verge, AJCC 8th Edition T3-4 N0-2, resectable disease and minimal duration of follow-up 1 year after radiotherapy.

We excluded patients with metastatic disease at staging and previous pelvic irradiation. Patients with contra-indication to MRI imaging, or in whom MRI-guided delineation was not feasible due to artefacts, were excluded. Patients unfit for chemotherapy as per multidisciplinary assessment were excluded. Patients were staged using endoscopy with biopsy, laboratory analysis including carcinoembryonic antigen (CEA), diagnostic MRI and computed tomography (CT) of the thorax and abdomen. In case of elevated CEA or ambiguous findings using CT, a complementary 18-fluorodeoxyglucose positron emission tomography (FDG PET) CT or MRI liver could be associated. All cases were discussed in the multidisciplinary tumor board (MTB).

### 2.2. Radiotherapy

Patients were treated adaptively on an MRI-based linear accelerator (MRIdian, ViewRay Inc., Denver, CO, USA). Radiotherapy consisted of 5 × 5 Gy on the mesorectum (Clinical Target Volume (CTV) with a simultaneous integrated boost (SIB) of 5 × 6 Gy on the GTV. The CTV was defined according to the 2016 international consensus guidelines on clinical target volume delineation in rectal cancer [8].

Patients were instructed to drink 250–300 mL of water 1 h prior to simulation and each treatment session. This is done to facilitate consistent and reproducible positioning of internal organs, particularly a full bladder during radiotherapy. For the boost planning target volume (PTV), an isotropic margin of 5 mm was used around the GTV.

PTV margins for the 25 Gy CTV were 11 mm anteriorly, 7 mm posteriorly, 8 mm laterally and 10 mm cranially and caudally. Cine-MRI gating was performed using the GTV with an isotropic margin of 3 mm as the gating structure (region of interest). If more than 5% of this region of interest fell outside the boundaries of the gating structure, an automatic beam-hold was performed.

### 2.3. Chemotherapy

At 10–18 days after the end of radiotherapy, the patient started chemotherapy, consisting of CAPOX (oxaliplatin 130 mg/m^2^ on day 1 and capecitabine 1000 mg/m^2^ twice daily on day 1–14 of a three-week cycle) or modified FOLFOX4 (oxaliplatin 85 mg/m^2^ day 1, folinic acid 800 mg day 1 and 5-fluorouracil 2400 mg/m^2^ in a continuous infusion over 46 h in a two-week cycle). The treatment duration was 18 weeks, i.e., 6 cycles of CAPOX or 9 cycles of FOLFOX. Dose reductions were performed as per standard of care.

### 2.4. Treatment Effect Assessment

Treatment effect assessment using flexible sigmoidoscopy, CT of the thorax and abdomen, and MRI of the rectum was planned at week 11–12 and week 23–24 after the start of the treatment. All cases were discussed in the MTB after each assessment, leading to a final evaluation around week 26, after which the final treatment plan was discussed with the patient.

### 2.5. Surgery

Surgery was planned 4–6 weeks after the last chemotherapeutic treatment cycle. The type of surgery was decided upon in the MTB. If, at the final assessment, patients achieved a complete clinical response (cCR) as defined by Maas et al., they were eligible to participate in a watch-and-wait protocol [9].

Follow-up within the watch-and-wait protocol consisted of rectal MRI, CT thorax/abdomen, CEA, clinical examination, digital rectal examination (DRE), and flexible sigmoidoscopy every three months of the first year, clinical and digital examination and CEA every 3 months and alternating rectal MRI and flexible sigmoidoscopy every 6 months of the second year. In year 3–5, a clinical follow-up is performed every 6 months with alternating rectal MRI and rectosigmoidoscopy every 12 months.

For patients who underwent surgery, follow-up consisted of clinical examination and blood tests, including assessment of carcinoembryonic antigen (CEA) levels, every 3 months, and CT imaging of the thorax and abdomen every 6 months during the first 2 years. Between years 3 and 5, patients underwent clinical examination and CEA assessment every 6 months, with annual CT imaging of the thorax and abdomen. Colonoscopy was performed 1 year postoperatively and subsequently every 3 years.

### 2.6. Pathological Assessment

The following variables were extracted from the pathology reports of patients who were operated: surgical resection specimen quality, number of lymph nodes examined, residual tumor classification, distance to distal margin (in patients with residual tumor), lymphovascular invasion, perineural invasion, tumor regression and pathological tumor stage (according to TNM 8th edition). Surgical resection specimen quality was assessed using the Quirke classification [10]. Tumor regression was assessed using the Dworak regression grading system [11].

### 2.7. Data Gathering and Analysis

Patient data were extracted from the electronic health record (EHR). We extracted age and sex, dates of radiotherapy, type of chemotherapy and any dose reductions. Outcome variables regarding recurrence are based on the last available patient contact in the EHR. Data regarding acute adverse events was scored according to Common Terminology Criteria for Adverse Events, 5th edition (CTCAE v5.0) based on retrospective analysis of the EHR. Only the highest grade observed for each adverse event was recorded. Data analysis was performed using R. Data cut-off was 25 September 2025.

### 2.8. Data Sharing Statement

Anonymized patient data and the data analysis source code used in this study can be shared upon request. Interested researchers should contact the corresponding author to obtain access. The sharing of these data will require the establishment of a data sharing agreement.

## 3. Results

Of the patients treated in our institution between July 2021 and January 2024, 28 patients were eligible for inclusion. One patient was lost to follow-up and therefore excluded from the analysis. Our patients were mainly male (81%), and a significant proportion of patients (33%) was aged 70 or older. The median age was 62 years old (Q1–Q3: 51–70). Most patients (81%) had a good performance score at the start of the treatment (Karnofsky performance status 90 or 100). The tumor location was evenly distributed over the high, mid and lower rectum. Twenty-two out of twenty-seven patients had at least one high-risk feature, such as cT4, cN2, mesorectal fascia involvement (MRF+), or extramural vascular involvement (EMVI+). Full patient characteristics are presented in Table 1.

**Figure A1:**
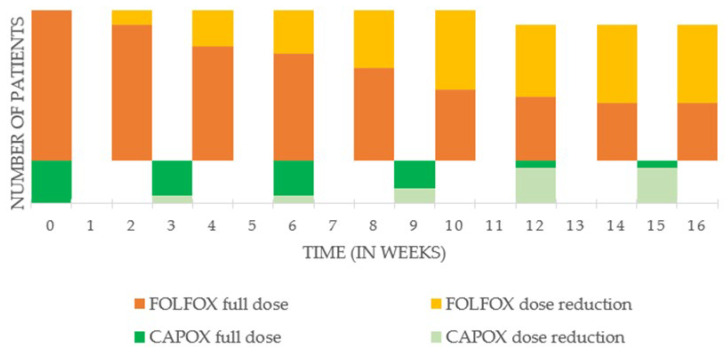
Dose compliance to preoperative chemotherapy. Proportion of patients treated with chemotherapy per course.

All patients completed radiotherapy as planned. Six patients received CAPOX, and 21 patients were treated with FOLFOX. Dose reductions of chemotherapy were necessary in 21 patients. Two patients did not complete the 18 weeks of chemotherapy as planned (details in Appendix A). Reasons for treatment discontinuation were grade 2 polyneuropathy and grade 3 cystitis with pararectal abscess formation. Figure A1 contains a more detailed visual representation of chemotherapy dose compliance. A complete assessment of treatment-emergent adverse events is listed in Table 2. The most prominent adverse events, scored either during radiotherapy or during chemotherapy, were paresthesia (*n* = 19, 70%), diarrhea (*n* = 10, 37%) and proctitis (*n* = 8, 30%). The most common severe adverse event was diarrhea, where eight patients (30%) experienced grade 2 or higher toxicity, followed by proctitis, with three patients (11%) experiencing grade 3 toxicity. No CTCAE grade 4 or 5 adverse events were observed.

**Figure 1:**
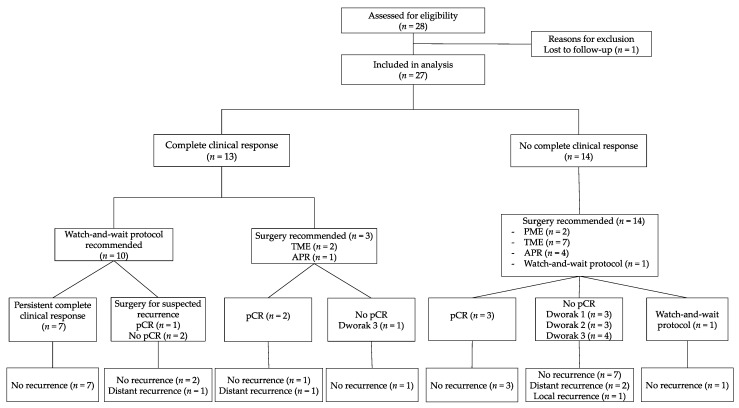
Study flow diagram.

The median follow-up was 39 months (or 3.25 years) (95% CI [35 (2.92 year); 45 (3.75 year)]) from the start of radiotherapy. Figure 1 shows the study flow diagram. Thirteen out of twenty-seven patients (48%) achieved a clinical complete response (cCR). Of these, 10 entered a watch-and-wait protocol for non-operative management (NOM). One patient with a near-complete response declined surgery, with an evolution to cCR, and joined the watch-and-wait protocol, with no recurrence observed on follow-up. This patient was not considered as a persistent clinical response in our analysis. Three out of eleven patients had suspected local regrowth and underwent salvage surgery. Pathological examination revealed a pathological complete response (pCR) in one patient and pathologically confirmed regrowth in the other two patients. One patient with local regrowth later developed distant metastasis (1 of 11).

Sixteen patients underwent surgery within 4–6 weeks after neoadjuvant treatment. Of these, nine underwent total mesorectal excision (TME), two had partial mesorectal excision (PME), and five received abdominoperineal resection (APR). One patient with a near-complete response declined surgery, with an evolution to cCR, and joined the watch-and-wait protocol, with no recurrence observed on follow-up. This patient was not considered as a persistent clinical response in our analysis. Among these patients, 37.5% (6/16) showed a pathological complete response (Dworak grade 4), and 18% (3/16) had no pathological response (Dworak grade 1). Table 3 provides a summary of surgical and pathological characteristics of all operated patients.

Of the patients with a complete pathological response, one patient (1 out of 5) had a distant recurrence which was treated with curative intent. Of the patients with no pCR (*n* = 11), local recurrence was observed in one patient and distant recurrence in two patients. One of the patients with distant recurrence died. Table 4 summarizes local control, distant relapse, and overall survival for the full cohort.

Regarding radiological assessment, post-treatment MRI showed clearance of involved MRF in seven out of 12 patients (58.3%). Among patients with extramural vascular invasion positivity on initial scan, EMVI status converted from positive to negative in 8/12 patients (66.7%).

## 4. Discussion

Although previous series of patients with rectal cancer treated with MRI-guided radiotherapy have been published, this is, to our knowledge, the first using total neoadjuvant short-course radiotherapy followed by chemotherapy [6,12,13].

Overall, the clinical and treatment characteristics of our cohort are representative of contemporary patients with locally advanced rectal cancer, although the proportion of male patients is somewhat higher than expected based on population registries. We also acknowledge that only 81% of patients in our cohort had at least one high-risk feature (as defined in the RAPIDO trial) [1].

Our results demonstrate that our approach with an integrated boost is feasible and tolerable. All patients were able to complete the radiotherapy as planned. Although 92% of patients completed the 18 weeks of chemotherapy, most patients (84%) required dose reductions. In further implementation of this treatment protocol, a reduction in chemotherapy duration from 18 to 12 weeks would be used as both appear to be equivalent in terms of oncological outcomes based on real-world data [14]. Our results appear encouraging, showing a persistent clinical complete response in 26% and pathological complete response in 22% of all patients. To put this into context, the pCR rate in RAPIDO was 24%, and a combined rate of 28% (persistent cCR and pCR) was observed in real-world data [3,14,15]. As stated in the introduction, analysis of the RAPIDO data seems to confirm that pCR is a predictor of local disease control in patients who have undergone total neoadjuvant therapy [3]. The increased rates of cCR (10/27) and pCR (6/27) with our approach could address the concerns of locoregional failure. This does not, however, address the increased breach of the mesorectum on the resection specimen observed in the intervention arm of RAPIDO, which is hypothesized to be the cause of increased local failure [2].

Of the 11 patients entering the watch-and-wait protocol, three patients (27%) had suspected regrowth. In only two patients was regrowth pathologically confirmed. This rate is similar to that observed in the larger LARCT-US cohort [14]. These three patients underwent R0 resection, and none had subsequent locoregional failure. Among the patients who proceeded to surgery 4–6 weeks after completing TNT, one developed unresectable locoregional recurrence.

The main limitations of our pilot study are the small patient number and retrospective character. In RAPIDO, the median time from surgery to the detection of locoregional recurrence was 21.6 months (interquartile range (IQR): 14.4–31.2) in the experimental arm and 14.4 months (IQR 9.6–32.4) in the standard treatment arm group [2]. Considering the 5–6-month treatment duration before the surgery typically takes place per protocol in RAPIDO, our median follow-up time of 39 months, counted from start of radiotherapy, seems sufficient to detect local recurrence, although additional follow-up could provide further insight. The low number of patients complicates the comparison of the primary outcomes with landmark trials. The rate of locoregional failure (4%) is on par with the control arm of RAPIDO (8%) and LARCT-US (6%). Distant recurrence was lower at 15% compared to 23% in RAPIDO and 24% in LARCT-US, where these same reservations apply.

Due to the retrospective nature of the study, documentation of adverse events is inherently less precise than in a prospective setting, especially for low-grade toxicities. Moreover, the lack of prospective registration compromises the accurate assessment of adverse event duration. Since chemotherapy timing overlaps the 90-day window for defining acute radiation-related adverse events, it is not possible to attribute the adverse events solely to radiotherapy or chemotherapy. We observed serious adverse events (CTCAE grade 3 or higher) in five patients. Neurological toxicity was the most frequent AE. Despite most cases being low-grade, oxaliplatin-induced neuropathy can significantly impact quality of life [16]. Importantly, although the detection and follow-up of low anterior rectal syndrome is standard practice in our institution, these data were not of sufficient consistency to be reported.

The spatial and temporal resolution of MRI provides the theoretical possibility to more precisely delineate the radiotherapy target compared to CT. However, uptake of this technique has been limited so far due to several factors, including cost implications and time constraints. It is difficult to ascertain the contribution of MRgRT over standard CT-guided radiotherapy.

Several phase II studies have combined dose-escalated SC-RT in a total neoadjuvant protocol. Chan et al. reported a phase II study on 76 patients with locally advanced rectal cancer using CT-guided SC-RT with a simultaneous integrated boost up to 5.5–6 Gy per fraction followed by four cycles of CAPOX. The pathological complete response was reported in 19% of patients. Furthermore, grade ≥ 3 surgical complications were observed in 10% of patients. Grade ≥ 3 toxicities related to the radiotherapy and/or chemotherapy were similar. Of note, the rate of proctitis was 34%, with 5% of patients reporting grade ≥ 3. This group reported a local recurrence rate of 6%, albeit with a shorter duration of follow-up than in our study (27 months) [17]. The prospective single-arm phase II SHORT-FOX study reported the use of a total neo-adjuvant regimen consisting of dose-escalated SC-RT (5 × 5 Gy with a sequential 5 Gy single fraction boost) followed by eight cycles of FOLFOXIRI. In 37 patients, 73% of whom had at least one high-risk feature, this regimen resulted in cCR and organ preservation in 25% of patients. Distant recurrence was observed in 21% of patients [18]. One of the possible explanations of the differences in pathological response observed is that MRI-guided radiotherapy improves target delineation, reducing the chance of geographical misses. It is, however, not possible to isolate the effect of the MRgRT or SIB from the overall treatment strategy in the absence of a control arm.

In the time between this trial and its publication, several pivotal studies have reshaped the treatment landscape of locally advanced rectal cancer. This has led to a decreased implementation of short-course radiotherapy in total neoadjuvant regimens. The PRODIGE23 trial demonstrated that treatment with six cycles of FOLFIRINOX followed by LC-CRT (experimental arm) versus LC-CRT (control arm) resulted in an improvement in the pathological complete response (28% vs. 12%) and disease-free survival (5y DFS 73% vs. 65%). Long-term follow-up reported an improvement in overall survival at 7 years (82% vs. 76%) [19,20,21]. The OPRA and ACO/ARO/AIO-12 trials explored LC-CRT followed by consolidation chemotherapy versus chemotherapy followed by LC-CRT preoperatively [22]. Furthermore, in the OPRA trial, non-operative management was possible in the event of a (near) clinical complete response [23]. LC-CRT followed by consolidation chemotherapy resulted in a higher number of patients with a pathological complete response with comparable disease-free survival (71% vs. 69%) [23]. This regimen is, at the time of writing, recommended in the 2025 ESMO guidelines as the preferred option when organ preservation is the treatment goal [24]. Furthermore, the results of the PROSPECT trial have shown that, in patients with T2-node-positive, T3-node-negative, or T3-node-positive rectal cancer, omitting LC-CRT in case of a good response following six cycles of FOLFOX chemotherapy was non-inferior in equal rates of disease-free survival (5y DFS 81% vs. 78%) [25]. Finally, the STAR TREC study reported at ESTRO 2025 showed higher rates of organ preservation at 1 year for LC-CRT (80%) over SC-RT (61%), although these data have not yet been published [26].

Despite this evolution in the field, our findings illustrate that a total neoadjuvant treatment with SC-RT remains relevant. Several recent studies pairing immune checkpoint inhibition (ICI) in the neoadjuvant setting for microsatellite stable rectal cancer have used short-course regimens. These regimens have reported preliminary positive results compared to standard LC-CRT [27,28]. A recent meta-analysis found a superior efficacy of the combination of ICI with SC-RT versus the combination with LC-CRT [29]. These findings are also supported by translational data [30]. A dose-intensified regimen with an integrated boost should be tested with this combination.

## 5. Conclusions

This retrospective analysis demonstrates that total neoadjuvant short-course MRI-guided radiotherapy with a simultaneous integrated boost followed by chemotherapy is a feasible and tolerable treatment approach for locally advanced rectal cancer. With a median follow-up of 39 months, nearly half of the patients achieved a persistent clinical or pathological complete response, exceeding rates reported in landmark trials like RAPIDO. The watch-and-wait strategy allowed for non-operative management in patients with complete clinical response, with acceptable rates of salvage surgery and locoregional failure. Although most patients required chemotherapy dose reductions, treatment completion rates were high, and toxicity was manageable. Limitations include the retrospective design and small sample size, warranting prospective validation. Overall, these findings support the potential of MRI-guided integrated boost regimens within total neoadjuvant therapy frameworks to enhance local control and organ preservation in rectal cancer. Although our findings are enticing, they should be regarded as hypothesis-generating, and controlled trials are needed before any definitive conclusions can be drawn.

## Figures and Tables

**Table 1:** Patient baseline characteristics.

Characteristic	Distribution
Age (in years)	
Median (Q1–Q3)	62 (50.5–70)
Mean (SD)	61.4 (12.9)
Min–Max	32–84
Elderly patients (Age ≥ 70 years)	9 (33%)
Gender	
Male	22 (81%)
Female	5 (19%)
Karnofsky performance status	
100	2 (7%)
90	20 (74%)
80	4 (15%)
70	1 (4%)
Clinical T stage determined by MRI	
cT3	18 (67%)
cT3a	3
cT3b	9
cT3c	4
cT3d	2
cT4	9 (33%)
cT4a	6
cT4b	3
Clinical N stage determined by MRI	
cN0	8 (30%)
cN1	2 (7%)
cN1a	0
cN1b	2
cN2	17 (63%)
cN2a	15
cN2b	2
Distance from the anal verge (cm)	
High rectum (10–15)	11 (41%)
Mid rectum (5–10)	8 (30%)
Low rectum (0–5)	8 (30%)
Extramural vascular invasion (EMVI+) determined by MRI	
No	15 (56%)
Yes	12 (44%)
Mesorectal fascia involvement (MRF+) determined by MRI	
No	15 (56%)
Yes	12 (44%)
High-risk feature	
No	5 (19%)
Yes	22 (81%)

**Table 2:** Treatment-emergent adverse events.

Adverse Event	Any Grade		Grade 1		Grade 2+		Grade 2		Grade 3	
	*n*	(%)	*n*	(%)	*n*	(%)	*n*	(%)	*n*	(%)
Paresthesia	19	(70%)	15	(55%)	4	(15%)	4	(15%)	0	-
Diarrhea	10	(37%)	2	(7%)	8	(30%)	7	(26%)	1	(4%)
Proctitis	8	(30%)	2	(7%)	6	(22%)	3	(11%)	3	(11%)
Platelet count decreased	5	(18%)	4	(15%)	1	(4%)	1	(4%)	0	-
Neutrophil count decreased	5	(18%)	2	(7%)	3	(11%)	2	(7%)	1	(4%)
Rectal pain	4	(15%)	1	(4%)	3	(11%)	3	(11%)	0	-
Alkaline phosphatase increased	3	(11%)	2	(7%)	1	(4%)	1	(4%)	0	-
Lipase increased	3	(11%)	0	-	3	(11%)	3	(11%)	0	-
Alopecia	2	(7%)	2	(7%)	0	-	0	-	0	-
Anal fistula	2	(7%)	0	-	2	(7%)	1	(4%)	1	(4%)
Abdominal pain	1	(4%)	0	-	1	(4%)	1	(4%)	0	-
Alanine aminotransferase increased	1	(4%)	0	-	1	(4%)	0	-	1	(4%)
Anemia	1	(4%)	1	(4%)	0	-	0	-	0	-
Anorexia	2	(7%)	1	(4%)	1	(4%)	1	(4%)	0	-
Constipation	1	(4%)	1	(4%)	0	-	0	-	0	-
Cystitis non-infective	1	(4%)	0	-	0	-	0	-	0	-
Hyperkalemia	1	(4%)	1	(4%)	0	-	0	-	0	-
Prolapse of intestinal stoma	1	(4%)	0	-	1	(4%)	1	(4%)	0	-
Urinary frequency	1	(4%)	0	-	1	(4%)	1	(4%)	0	-

**Table 3:** Surgical and pathological characteristics of operated patients.

	Number of Patients	(%)
Type of surgery	(*n* = 19)	
PME	3	(15.8%)
TME	10	(52.6%)
APR	6	(31.5%)
Dworak regression score	(*n* = 19)	
1	4	(21.0%)
2	4	(21.0%)
3	5	(26.3%)
4	6	(31.6%)
Residual tumor classification	(*n* = 19)	
R0 > 1 mm	18	(94.7%)
R1 ≤ 1 mm	1	(5.2%)
R2	0	(0.0%)
Circumferential resection margin	(*n* = 19)	
>1 mm	18	(94.7%)
≤1 mm	1	(5.2%)
Lymphovascular invasion	(*n* = 19)	
Yes	4	(21.0%)
No	15	(78.9%)
Perineural invasion	(*n* = 19)	
Yes	2	(10.5%)
No	17	(89.4%)
Distance to distal margin (in mm)	(*n* = 13)	
Median (range)	30 (4–60)	NA
Pathological T stage	(*n* = 19)	
ypT0	7	(36.8%)
ypTis	1	(5.3%)
ypT1	0	(0.0%)
ypT2	3	(15.8%)
ypT3	6	(31.6%)
ypT4	2	(10.5%)
Pathological N stage	(*n* = 19)	
ypN0	13	(68.4%)
ypN1	6	(31.6%)
ypN2	0	(0.0%)
Quality of TME	(*n* = 19)	
Complete	14	(73.7%)
Nearly Complete	5	(26.3%)
Incomplete	0	(0.0%)
Lymph node count on resection specimen	(*n* = 19)	
≥12	12	(63.2%)
<12	7	(36.8%)

NA: Not Applicable.

**Table 4:** Outcome parameters.

	Full Dataset (*n* = 27)	Non-Operative Management (*n* = 11)	Surgical Treatment (*n* = 16)
Locoregional failure			
No	26 (96%)	11 (100%)	15 (94%)
Yes	1 (4%)	0 (0%)	1 (6%)
Distant relapse			
No	23 (85%)	10 (91%)	13 (81%)
Yes	4 (15%)	1 (9%)	3 (19%)
Overall survival			
No	1 (4%)	-	1 (6%)
Yes	26 (96%)	11 (100%)	15 (94%)

## Data Availability

The raw data supporting the conclusions of this article will be made available by the authors on request. Interested researchers should contact the corresponding author to obtain access. The sharing of these data will require the establishment of a data sharing agreement. All data sharing activities must comply with the Belgian data protection regulations as outlined in the Belgian Data Protection Act of 30 July 2018 and the European General Data Protection Regulation (GDPR) (Regulation (EU) 2016/679). These regulations ensure the protection of personal data and privacy of individuals within the European Union and the European Economic Area.

## Abbreviations

The following abbreviations are used in this manuscript:

AJCC	American Joint Committee on Cancer
CAPOX	Capecitabine and Oxaliplatin
APR	Abdominoperineal Resection
cCR	Clinical Complete Response
CEA	Carcinoembryonic Antigen
CI	Confidence Interval
CRM	Circumferential Resection Margin
CRT	Chemoradiotherapy
CT	Computed Tomography
CTCAE	Common Terminology Criteria for Adverse Events
CTV	Clinical Target Volume
DFS	Disease-Free Survival
DRE	Digital Rectal Examination
ECOG	Eastern Cooperative Oncology Group
EHR	Electronic Health Record
EMVI	Extramural Vascular Involvement
FDG	18-Fluorodeoxyglucose
FOLFIRINOX	5-Fluorouracil, Leucovorin, Irinotecan, and Oxaliplatin
FOLFOX	5-Fluorouracil, Leucovorin, and Oxaliplatin
GTV	Gross Tumor Volume
HR	Hazard Ratio
ICI	Immune Checkpoint Inhibitor
IQR	Interquartile Range
LARCT	Locally Advanced Rectal Cancer Trial
LC-CRT	Long-Course Chemoradiotherapy
MRI	Magnetic Resonance Imaging
MRgRT	MRI-guided radiotherapy
MTB	Multidisciplinary Tumor Board
NOM	Non-Operative management
pCR	Pathological Complete Response
PET	Positron Emission Tomography
PME	Partial Mesorectal Excision
PTV	Planning Target Volume
RT	Radiotherapy
SC-RT	Short-Course Radiotherapy
SIB	Simultaneous Integrated Boost
TME	Total Mesorectal Excision
TNT	Total Neoadjuvant Therapy

## Appendix A

Figure A1 provides a visual representation of dose compliance during chemotherapy treatment. After six cycles of FOLFOX or four cycles of CAPOX, most patients required dose reductions, mainly of oxaliplatin. The main reason for dose reduction was peripheral neuropathy. Two patients did not complete all 18 weeks of treatment.
